# Debulking corneal biopsy with tectonic amniotic membrane transplantation in refractory clinically presumed fungal keratitis

**DOI:** 10.1038/s41598-023-50987-4

**Published:** 2024-01-04

**Authors:** Taher K. Eleiwa, Gehad H. Youssef, Ibrahim Abdelkhalik Elsaadani, Samar N. Abdelrahman, Ahmed A. Khater

**Affiliations:** 1 Department of Ophthalmology, Benha Faculty of Medicine, Benha University Hospitals, Benha University, Al-Sahaa Street, Diverted From Farid Nada St., Benha, 13511 Egypt; 2 Department of Clinical Pathology, Benha University Hospitals, Benha University, Benha, Egypt

**Keywords:** Microbiology, Diseases, Health care, Medical research

## Abstract

The treatment of fungal keratitis (FK) is challenging due to the subacute indolent course, and initial misdiagnosis. In this retrospective case series, we highlight both the diagnostic and therapeutic roles of corneal biopsy together with amniotic membrane transplantation (AMT) in patients with refractory clinically presumed FK. Debulking biopsy and tectonic AMT were performed during the initial presentation. Biopsy specimens were sent for KOH smears and cultures. After KOH smears confirmed the presence of fungal elements, topical voriconazole 1% was prescribed for the first 72 h then tailored according to the clinical response and the culture results. The outcome measures were complete resolution of infection and restoration of corneal integrity. Cases associated with culture proven bacterial keratitis were excluded. Twelve cases were included in the study. KOH smears confirmed the presence of fungal growth in all specimens. Cultures grew Aspergillus in 6/12 cases, sensitive to voriconazole (5/6) and amphotericin (3/6); Fusarium (4/12), sensitive to both voriconazole and amphotericin; and no growth in 2/12 cases. Amphotericin 0.15% eye drops were added to the 7 cases with proven sensitivity and to the remaining 2 culture negative cases. Gradual resolution of infection was seen in all cases after 35.6 ± 7.8 days. In FK, a debulking biopsy simultaneously with AMT help decrease the microbial load, suppress the inflammatory process, support the corneal integrity, confirm the presence of fungal pathogen.

## Introduction

Fungal infection of the cornea is one of the serious causes of microbial keratitis that can lead to sight-threatening complications and catastrophic sequelae if not correctly diagnosed and managed^1,2^. Fungal keratitis (FK) represents fewer than 10% of the infectious keratitis burden in developed countries versus 40% in developing countries with tropical climates^3–6^. In China, FK was found to account for up to 62% of severe microbial keratitis and was recognized as a frequent indication of keratoplasty^7^. Among the widely known species of fungi, molds, including Aspergillus and fusarium species, are the most encountered in patients with FK. Different modes of infection have been described, with the trauma of plant origin or organic matter being among the most familiar routes of acquiring the disease, mainly in developing countries^6,8^. A tropical climate, chronic use of topical steroids, chronic ocular surface disorders, and diabetes mellitus are other risk factors for FK. Also, fusarium species are ubiquitous in the surrounding environment, and the infection could be acquired via direct contact with fusarium-contaminated substances^5,9, 10^.

Diagnosis of FK is notoriously challenging and usually delayed due to the indolent course of the disease. Hence, patients could present with a descemetocele, corneal perforation, or intraocular spread of infection^11,12^ It is noteworthy that the patient may just complain of minimal ocular irritation in the presence of a severely infiltrated cornea^13^. Several invasive and non-invasive ways have been reported to aid the diagnosis of FK. Non-invasive methods include in vivo confocal microscopy (IVCM), which can visualize the pathogen in the corneal tissue. However, being expensive and needing expert interpretation have limited its use in routine clinical practice^14^. Invasive methods, which are more frequently used, including direct examination of corneal scrape or biopsy materials with 10% potassium hydroxide (KOH) smears under a light microscope for instantaneous detection of the fungal hyphae with an 80–99.3% sensitivity and an 83.8–99.1% specificity^15^. Also, polymerase chain reaction (PCR) has been reported as a fast diagnostic tool with small amounts of corneal tissue. However, the cost and requirement of a well-equipped diagnostic lab limited its routine clinical use^16^. Culture on different media, including Sabouraud dextrose agar (SDA), blood agar, and brain heart infusion broth, to detect the fungal spores. However, cultures take longer, which may delay the diagnosis, making the KOH smears a preferable primary approach for diagnosing patients with suspected FK^17^. False positive results, owing to contamination and non-pathogenic bystanders, have been reported. Thus caution must be given while interpreting culture results, especially if they are not concordant with the clinical findings^14^.

Treating FK can be a real challenge, especially in resistant cases. The first treatment line is topical antifungal agents, most of which are fungistatic agents like polyenes, including natamycin and amphotericin B, and azoles, including fluconazole and voriconazole*.* The ideal antifungal drug should have high efficacy with good penetration and be non-toxic to the corneal tissue. The fungistatic nature of topical antifungals and the poor penetration into deeper corneal tissue make them insufficient for eradicating the infection, especially in deeper corneal lesions. Usually, another route of administration and/ or an adjuvant treatment line is needed to protect the corneal integrity and improve visual outcomes^18^. Intrastromal and intracameral routes can be used to increase drug delivery^19^. Therapeutic penetrating keratoplasty (TPK) may be required, as a globe salvage procedure, in cases with corneal melting or perforation, albeit with a high risk of reinfection, rejection, and graft failure^20^.

Limited literature is currently available for using AM in cases of microbial keratitis, including those caused by fungal pathogens. Only a few reports have tested the efficacy and safety of AM Ref.^21^ as an adjuvant treatment option for cases with resistant FK (Table 1). In this retrospective case series, we report the outcomes of simultaneous debulking corneal biopsy and tectonic AMT in refractory clinically presumed FK.

**Table 1:** Summary of reports for using amniotic membrane transplantation in fungal keratitis.

Study	Description	Epithelial healing rate	The necessity of therapeutic penetrating keratoplasty (TPK)	Persistence of infection	Visual acuity	Complications
Chen et al.^22^	• 23 eyes of 23 patients (69.6%: active phase, 30.4%: non-active) • Follow up: (6–65) months • Single-layer AMT: 17 cases • Double-layer AMT: 6 cases (perforated), both with epithelial side. up	Complete healing in 12 cases (75%) of the active group and seven patients (100%) in the inactive group	Four patients (25%) in the active group	Two patients (from those who underwent TPK)	The final BCVA improved in 17 cases, worsened in 2 patients, and remained unchanged in 4 cases	AM melt in 3 cases
Qing et al.^23^	• 210 eyes of 210 patients (Group1 (109 eyes): AMG + antifungal + debridement) versus group 2 (101 eyes): debridement + antifungal)	The therapeutic course was (22.4 ± 1.8) days in group 1, significantly shorter than in group 2 (33.2 ± 2.4)			Postoperative visual acuity was better in group 1	
Zeng et al.^24^	• 20 eyes of 20 patients (group 1: AMG + antifungal + debridement, 10 eyes) **versus group 2:** debridement + antifungal; 10 eyes)	The average healing time of the AM group was 6.89 ± 2.98 days, which was shorter than that of the other group (10.23 ± 2.78 days)	1 case from group 1 Vs. 2 in group 2 needed TPK		The average UCVA of the AM group was 0.138 ± 0.083, which was statistically better than that of the other group (0.053 ± 0.068)	
Kim et al.^25^	• Case series: 21 eyes of 21 patients with infective keratitis (2 cases FK) • Follow-up (4–28) months	Complete healing in + /− 10 days	1 case performed PKP		It improved post-op BCVA in the 2 cases One case improved from LP to 14/20 after performing PK	
Qiong et al.^26^	• 41 eyes of 41 patients with culture-proven fusarium keratitis • All cases: focal keratectomy + AMT • Group 1, 22 eyes (+ cryotherapy) versus group 2, 19 eyes (no cryotherapy) • Follow up (3–12) months	Faster healing in group 1				
Abdulhalim et al.^27^	• 40 eyes of 40 patients (12 eyes with fungal keratitis received conjunctival flap versus 13 with fungal keratitis received AMT) • Follow up: 6 months	No significant differences between the two groups			No significant differences between the two groups	
Eleiwa et al.^28^	• Case series: 5 patients with post-LASIK fungal keratitis • (They underwent intrastromal injection of Amphotericin B, LASIK flap amputation, and AMT) • Follow-up: 12–18 months	26 ± 1.8 (median, 27; range, 22–32) days	All cases had elective PKP done 2.4 (median, 2; range, 2–3) months after AMT		The final BCVA ranged from 20/20 to 20/80 at an average follow-up of (median, 14; range, 12–18) months	

*AMT* Amniotic membrane transplantation, *BCVA* best corrected visual acuity, *TPK* therapeutic keratoplasty, *PKP* penetrating keratoplasty.

## Methodology

We conducted a hospital-based interventional 2-year retrospective case series that was approved by the Institutional Review Board of Benha University hospital and met the Declaration of Helsinki. Cases with refractory clinically presumed fungal keratitis were included. Clinical predictors of fungal keratitis were satellite lesions, deeper lesions, stromal melting, endothelial plaque, longer duration since onset and history of trauma^22^. Predictors of corneal perforation were presence of hypopyon, large infiltrate size and infiltrate involving the posterior third of the cornea regardless of the type of the fungal pathogen^23^. Polymicrobial and other microbial keratitis were excluded.

Prior to surgery, informed consent was obtained from all patients. Human AM was prepared and preserved as previously reported^24^. We did the procedure under peribulbar anesthesia. All discharge and loose epithelium were removed. Povidone iodine in the conjunctival sac was avoided not to compromise the culture results. Starting in the healthy cornea, 1–2 mm away from infiltrates, a deep anterior lamellar keratectomy was performed as a debulking biopsy. Briefly, a small crescent blade was used to cut into the deeper layers of the cornea, while holding the edge with a micro-notch forceps, till the deep layer of the infiltrate was reached. The biopsy specimen was subdivided and sent immediately for KOH smears, histopathological examination and cultures. At the same setting, adjuvant tectonic AMT was performed. AM, priorly soaked in voriconazole 1%, was cut into multiple pieces and fashioned slightly smaller to the post-debulking defect size, then layered epithelium-side-down to fill the facet. Then, a larger piece of AM was trimmed and sutured, as a patch graft, in an epithelial-side-up position with a running/interrrupted 10–0 nylon and not overlapping with the edge of the epithelial defect. On confirming the presence of fungal pathogen via the KOH smears, a compounded topical antifungal was prescribed hourly, and the corneal response was monitored daily till the culture results. Initial modification of treatment, before culture results, was planned when suboptimal response was observed by adding/switching the antifungal medication. The antifungal choices were either voriconazole 1% and/or amphotericin B 0.15%^25^.

The antifungal drug sensitivity tests were conducted using disc diffusion methods in adherence to the National Committee for Clinical Laboratory Standards (NCCLS) guidelines^26^. The control strain utilized was Candida albicans (ATCC 10231). The co-author (S.A) employed seven types of antifungal discs, including natamycin, voriconazole, amphotericin B, micronazole, fluconazole, ketoconazole, and itraconazole. Interpretation of clearance zone diameters was conducted in accordance with the guidelines proposed by the manufacturer.

## Results

**Figure 1:**
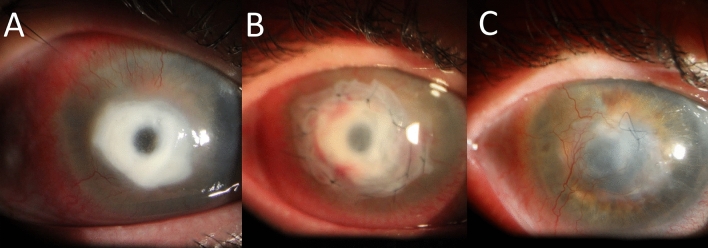
(**A**) Case #12 presented with clinically presumed fungal keratitis with central descemetocele, and (**B**) had a corneal biopsy with tectonic amniotic membrane transplantation (**B**). (**C**) Slit-lamp photo showing complete corneal healing with a central leucoma non-adherent after 45 days from the surgery and topical voriconazole 1%.

We report the clinical course of 12 eyes of 12 patients with refractory keratitis that met the clinical criteria of FK and underwent corneal debulking biopsy and a tectonic AMT. Table 2 elaborates the clinical criteria of study cases. The mean age of the patients was 50.5 ± 9.3 (median, 49; range, 36–67) years. Time between initial symptoms and referral to our hospital was 25.8 ± 10.2 (median, 22.5; range, 14–42) days. All patients were prescribed topical antibiotics, and only 5 patients were also given commercially available antifungal natamycin drops. However, cases #2, #11 and #12 were initially misdiagnosed as viral necrotizing stromal keratits and treated with topical prednisolone acetate 1%, preservative-free lubricants and systemic valacyclovir. The presenting vision, corneal signs, and prior eye drops are summarized in Table 2. KOH smears revealed fungal hyphae in all specimens. No empirical intrastromal or periocular or intracameral injections were done throughout the course of treatment. Surgeries were done uneventfully in all cases. Case #12 had iatrogenic microperforations during keratectomy that were sealed successfully with AMT (Fig. 1). The Sabouraud’s agar grew Aspergillus in 6/12 cases, sensitive to voriconazole (5/6) and amphotericin (3/6); and Fusarium (4/12), sensitive to both voriconazole and amphotericin. No growth was detected in 2/12 cases. Compounded amphotericin 0.15% eye drops were added to the 7 cases with proven sensitivity and to the remaining 2 culture negative cases. Complete resolution of infection, re-epithelialization of corneal surface, and restoration of corneal thickness were seen within 35.6 ± 7.8 (median, 36; range, 23–45) days in all cases. Topical treatment was gradually tapered after full resolution of infection. Elective optical PK was performed uneventfully in 10 eyes 4.5 ± 0.9 (median, 5; range, 3–6) months later.

**Table 2:** Characteristics of the study cases.

Case	Age (years)	Time from onset of symptoms till referral (days)	Prior medications	Presenting visual acuity	Corneal signs	KOH smear	Culture results	Time to healing (days)	Time from healing to penetrating keratoplasty (months)
1	67	42	Voriconazole 1%, Tobramycin drops	1/60	whitish corneal stromal infiltrates with feathery margins	Fungal hyphae	Fusarium	44	5
2	53	28	Moxifloxacin and Natamycin drops	Hand motion	central corneal infiltration with hypopyon	Fungal hyphae	Aspergillus	35	4
3	75	23	Gatifloxacin 0.5% drops	Hand motion	focal stromal infiltrate with a heaped-up hypopyon	Fungal elements	Aspergillus	31	Scheduled
4	36	14	Ciprofloxacin drops and Natamycin drops	3/60	Paracentral focal stromal infiltration	Fungal hyphae	Fusarium	26	5
5	41	18	Neomycin-polymixin drops	Counting fingers 0.5 m	Hypopyon ulcer with endothelial plaque	Fungal hyphae	Aspergillus	33	3
6	57	22	Gatifloxacin 0.5% drops	Hand motion	Ring abscess with satellites	Fungal hyphae	Aspergillus	45	6
7	48	19	Natamycin drops	6/60	Central corneal ulcer with feathery extensions	Fungal hyphae	Fusarium	23	5
8	47	39	Moxifloxacin and Natamycin drops	1/60	Central focal stromal inflitrates	Fungal hyphae	No growth	29	3
9	50	16	Tobramycin drops, and Diflucan drops	Hand motion	Ring abscess with hypopyon	Fungal hyphae	No growth	43	5
10	61	23	Gatifloxacin 0.5% drops	2/60	Central corneal infiltration with gritty sensation on scrapings	Fungal hyphae	Aspergillus	37	4
11	45	37	Natamycin and Moxifloxacin drops	Hand motion	Fluffy corneal infiltrates with sanguineous hypopyon	Fungal hyphae	Fusarium	41	5
12	56	56	Topical Diflucan and natamycin	Hand motion	Corneal abscess and central Descemetocele and sanguineous hypopyon	Fungal hyphae	Aspergillus	45	Scheduled

## Discussion

The treatment of FK is intriguing as symptoms are subtle, and keratitis is minimal early in the clinical course. Initial misdiagnosis and partial response to empirical topical antibiotics and steroids, along with the typically slow progression, have made the clinical diagnosis challenging as well as the referrals when complicated. Having clinical data regarding the onset and the stage of keratitis is crucial to interpret the results of microbiological workup. It is also known that fungi can proliferate and spread through the corneal channels after gaining access through the corneal epithelium. Without releasing chemotactic factors, fungi can colonize corneal tissue while delaying the host immune response. Further, being immune privileged with limited defense mechanisms, the cornea can be easily colonized by fungi yielding symbiotic germination that is required to promote fungal pathogenicity and drug resistance^27^. Debulking corneal biopsy has both diagnostic and therapeutic roles in refractory infectious keratitis, particularly those with progressive stromal infiltrates inaccessible to corneal scrapings^28,29^. In our series, we had our biopsy specimens examined histopathological and cultures. Pathological examination was positive in all cases, and cultures were positive in 83%. When compared to corneal scraping, biopsy possesses the benefit of extracting samples from the deeper layers of the cornea and/or reducing the infection’s size, resulting in an overall diagnostic success rate up to 82%^28,30^. Moreover, according to Younger et al., histopathologic examination of corneal biopsies demonstrated a higher rate of microorganism identification compared to culture alone (40% vs. 19%)^31^. Being inexpensive, fast, sensitive, and specific, we relied mainly on the KOH smear^32^ to start the antifungal eye drops immediately after the surgery. The common practice in identifying fungi in infectious keratitis cases involves using 10% KOH smears, which have an overall sensitivity ranging from 81 to 99%^33^. Similarly, KOH demonstrates a high sensitivity in detecting Acanthamoeba (84–91%)^33^ and microsporidia (97%)^34^. Other quick tools, such as PCR, were unavailable in our department. It is also essential to recognize false positive and false negative culture results if not consistent with the clinical presentation. Even though the culture results are of prime importance to identify the fungus species and to judge the fungus sensitivity to various antifungal drugs, direct examination of the biopsy specimens, using KOH, is considered superior in the diagnosis of FK^29^.

Although corneal biopsy is a valuable diagnostic procedure, it is crucial to acknowledge that it carries certain risks. There have been reported cases of inadvertent corneal perforation associated with biopsy, particularly when there is pre-existing corneal thinning, melting, and necrosis. These conditions can create a misleading appearance of a thick and swollen cornea. Thus, tectonic AMT was performed simultaneously in our case series to support the thinned residual corneal stroma, and seal inadvertent perforations. Table 1 summarizes seven reports on using AM in FK. AMT is well-known to have triple antimicrobial, anti-inflammatory, and tectonic properties. First, the antimicrobial activity is attributed to the existence of lysozyme, immunoglobulin, and transferrin in the amniotic fluid^35^. Second, regulation of T-cell function and secretion of an anti-inflammatory agent such as IL-1ra, sTNF, and VEGF-R constitute anti-inflammatory activities of the AM that also exhibit a long-term beneficial effect on the stability of corneal surface and reduce the severity and density of neovascularization^36,37^. Third, AM has shown to be aggressively repopulated by cornea stroma-derived cells, ultimately restoring corneal stromal integrity and helping obviate the necessity of emergent therapeutic keratoplasty in cases with impending or actual corneal perforation^38,39^. In the context of infectious keratitis, corneal transplantation has a high risk of rejection, reinfection, and ultimately failure. The AM is considered an inexpensive and effective alternative to tectonic therapeutic keratoplasty in cases of corneal melting and impending perforation^40^. Adequate debridement of necrotic corneal tissue and radical debridement of the corneal infiltrates help decrease the microbial load; nevertheless, it makes the cornea vulnerable to perforation. Hence, the AM provides invaluable tectonic support and helps maintaining corneal integrity during the initial postoperative period while waiting for the culture results. Together with corneal biopsy, we did AMT during the initial presentation to help start the antifungal drops as soon as possible. It is well established that early AMT helps accelerating corneal healing and improve visual outcomes in fungal and bacterial keratitis. Additionally, soaking the AM in antibiotics before transplantation sustains drug delivery during the early postoperative period^41^. Meanwhile, AM helps reducing the potential epithelial toxicity of compounded topical medications such as amphotericin B. In our cases, a reduction of the clinical severity of keratitis with subjective improvement was noted in the early postoperative period. This could be explained by the radical debridement therapeutic effect and AM's aforementioned inhibitory effect on endogenous and fungal proteinase activities. Successful corneal surface reconstruction and resolution of infection were achieved in our cases without complications.

Our study is not without limitations. First, we did not include other forms of microbial keratitis. It is noteworthy to mention that filamentous fungal keratitis is the predominant microbial keratitis in the Egyptian Delta region where the study was conducted^42^. Nevertheless, further studies are required to evaluate the efficacy of the aforementioned intervention in polymicrobial and non-fungal keratitis. Second, our results should be interpreted with caution given the small sample size, lack of control group and the retrospective design. Further prospective clinical trials with larger sample sizes are required to replicate our findings.

In conclusion, we recommend performing a debulking biopsy with tectonic AMT in refractory clinically presumed FK to decrease the microbial load, confirm the presence of fungal pathogen, suppress the inflammatory process, restore the stromal integrity, and promote epithelial healing.

## Data Availability

All data generated or analyzed during this study are included in this published article.

## Abbreviations

AMT: Amniotic membrane transplantation
BCVA: Best corrected visual acuity
FK: Fungal keratitis
IVCM: In vivo confocal microscopy
KOH: Potassium hydroxide
MMP: Matrix metalloproteinases
PCR: Polymerase chain reaction
SDA: Sabouraud dextrose agar
TKP: Therapeutic keratoplasty
NCCLS: National Committee for Clinical Laboratory Standards
